# A Case Study on the Implementation of a Positive Youth Development Program (Project P.A.T.H.S.) in a Changing Education Policy Environment

**DOI:** 10.1100/tsw.2008.122

**Published:** 2008-10-10

**Authors:** Tak Yan Lee

**Affiliations:** Department of Applied Social Studies, City University of Hong Kong, China

**Keywords:** adolescence, positive youth development, education reform, liberal studies, school policy, project implementation, Project P.A.T.H.S.

## Abstract

This investigation of the implementation of a positive youth development program (Project P.A.T.H.S.) was part of a large study undertaken comprehensively to explore how effective the Tier 1 Program was in practice and how the results can shed light on future developments. Case studies on randomly selected schools were conducted in order to examine the factors that influence the process and quality of implementation of the Tier 1 Program of the Project P.A.T.H.S. Through interviews with the school contact person and focus group interviews with the teachers, an integration of the findings of these studies showed that five factors related to the program, people, process, policy, and place (5 “P”s) facilitated the implementation process of the Tier 1 Program in the school. Based on the integrated findings of a randomly selected school, it was found that the school made use of the changes in the educational policy environment to facilitate school policy and structural changes, to pave the way for the success of the implementation of a new and “unfamiliar” curriculum. Overall, the quality of program implementation in the school was good and the program was well received by the program implementers. Implications of the present findings for future program implementation with reference to school administrative arrangements and implementation issues are also discussed.

## INTRODUCTION

### Background

In response to the overwhelming trend of globalization and its potential impacts on a fragile economy, Hong Kong has gone through a decade of education reform[1]. This reform was launched in 1997 as part of a high-flying plan to lead Hong Kong into a new era. Since then, some fundamental changes have been made, notably the new 3-year senior secondary education, a reform of the academic structure, and strengthening of language teaching[2].

The education reform in Hong Kong was launched partly because of the observation that many students who performed well in academic subjects were weak in various abilities, including moral competency[3]. In the past, the moral education of Hong Kong was not as well structured as the academic curriculum. In contrast to a top-down model, the global trend focuses on teaching students how to think morally, discern moral issues, and handle moral problems. In the face of this situation, there is a call for a balanced development between the intellectual mind and the ethical mind for our young people.

## Education Reform

Over the past 10 years, the Government of the Hong Kong Special Administrative Region of the People's Republic of China has increased the expenditure on education by 40.5%. In the 2007/08 budget, education accounted for 21.4% of the total government expenditure – the largest single item[4]. Yet, the decade of education reform brought controversies and tensions between the education authority and major stakeholders, especially teachers and school-sponsoring bodies. The pace of new initiatives introduced was criticized as being hasty. Legislators passed a motion in 2006 urging the government to take all necessary steps to reduce the workload and ease the pressure on teachers. Although the officials in charge of educational policy explained that a new policy was well paved after a preparatory period of 3–4 years, the quantity and magnitude of changes in this decade was considerable. Among these changes, the reform of the academic structure (new 3-year senior secondary education, NSS) and the introduction of a new subject, Liberal Studies, had extensive implications. The NSS Report[5] stipulated that Liberal Studies will be assessed as a core subject in the 3-year senior secondary curriculum.

## Policy Change and Curriculum Reform

To be implemented in September 2009, the characteristics of the NSS include the provision of a broad and balanced curriculum; the replacement of the traditional practice of streaming students into science, arts, and commercial subjects; the provision of more choices in subjects; and a new requirement for all students to take Liberal Studies, on top of three core subjects: Chinese Language, English Language, and Mathematics. These four core subjects will take up 45–55% of the total curriculum. Students will be allowed to take two to three Elective Subjects or courses in Applied Learning. This part will only take up 20–30%. To better streamline the tradition of examination-orientated senior secondary levels, students will be required to be exposed to "Other Learning Experiences", which include moral and civic education, community service, aesthetic and physical experiences, and work-related experiences. This part will take up about 15–35%[5].

## Liberal Studies as a Core Subject

One of the major goals of education reform in Hong Kong is to nurture responsible citizens through self-understanding, critical thinking, and whole-person development. In this light, Liberal Studies provides opportunities for students to explore issues relevant to human conditions, enables students to make connections among different disciplines, and examines issues from a variety of perspectives so as to prepare students for the globalized economy.

There are six modules in Liberal Studies. They are grouped into three areas: (1) Self and Personal Development, which includes Module 1 Personal Development and Interpersonal Relationships; (2) Society and Culture, covering three modules: Hong Kong Today, Modern China, and Globalization; and

(3) Science, Technology, and the Environment, which includes two modules: Public Health, and Energy Technology and the Environment. The aims of Liberal Studies at the senior secondary level are sixfold[6].

1. To enhance the students' understanding of themselves, their society, their nation, the human world, and the physical environment
2. To enable students to develop multiple perspectives on perennial and contemporary issues in different contexts (e.g., cultural, social, economic, political, and technological contexts)
3. To help students become independent thinkers so that they can construct knowledge appropriate to changing personal and social circumstances
4. To develop in students a range of skills for lifelong learning, including critical thinking skills, creativity, problem-solving skills, communication skills, and information technology skills
5. To help students appreciate and respect diversity in cultures and views in a pluralistic society and handle conflicting values
6. To help students develop positive values and attitude towards life, so that they can become informed and responsible citizens of society, the country, and the world

Kwok[7] argued that there is an urgent need to offer Liberal Studies in Hong Kong because it is an important part of whole-person education in contemporary society. At the same time, it also balances the impact of specialized education. Preparation for NSS Liberal Studies involves the students' acquisition of foundation knowledge, sound development of generic skills, and positive values and attitudes in the previous key stages. Secondary schools are required to examine their curriculum initiatives to check whether such NSS Liberal Studies related knowledge, skills, and values are acquired by junior secondary students. According to the Education Bureau, "the appreciation and respect for diversity in cultures and views and the development of positive values and attitudes towards life, while being aims of New Senior Secondary Liberal Studies, should also exist in the learning experience of S1-S3 students"[8].

Under this new policy environment, secondary schools in Hong Kong had to take curriculum initiatives to meet this need. There were various ways to cope with the policy change starting in 2006. A school might not offer Liberal Studies in Junior Secondary, but might implement Integrated Humanities, a school-based curriculum that combined humanities subjects including History and Geography, as well as Economic and Public Affairs. Through this subject, students were expected to understand the interrelationship of knowledge and learn to think from different perspectives on given issues. Yet another school might provide chances for students to discuss issues concerning self-image, healthy lifestyle, self-management, and interpersonal relationships, in addition to providing knowledge needed for Module 1 in Liberal Studies in the class teacher's period, assemblies, or a special subject called Civic Education or Life Education. Some schools put the curriculum contents into Religious Studies.

## The Development of a Positive Youth Development Program

Similar to their counterparts, adolescents in Hong Kong are inevitably subject to moral issues and dilemmas; for example, the challenge of drug abuse in rave parties and public entertainment places, violating intellectual property rights while surfing on the Internet, and the unrestrained influence of the media that creates a demoralizing effect as a result of their undue exposure to the sensationalism, violence, bloodshed, and pornography through day-to-day encounters. All of these pose an array of moral challenges to our youngsters who may feel lost, confused, and bewildered, given the many conflicting values.

In order to equip our future generations with the ability to exercise critical thinking and informed moral judgment so that they may harness changes with times and emerge as independently minded and responsible moral beings ready to contribute to the betterment of society, the nation, and the world at large, education, by common consent, provides the answers to these challenges.

"P.A.T.H.S. to Adulthood: A Jockey Club Youth Enhancement Scheme" is a holistic positive youth development program developed for junior secondary school students in Hong Kong. The word "P.A.T.H.S." denotes **P**ositive **A**dolescent **T**raining through **H**olistic **S**ocial Programmes[9,10], which has two tiers. While the Tier 1 Program is a universal program that utilizes a curricula-based approach for all Secondary 1 to 3 students of the participating schools, the Tier 2 Program is provided for students who have greater psychosocial needs.

Since the curriculum contents of the Project P.A.T.H.S. cover the core areas and extend well beyond the Module 1: "Personal Development and Interpersonal Relationships" in Liberal Studies of the NSS curriculum, the Project is well received. Furthermore, for the Tier 1 Program, each participating school has the freedom to determine who will teach the program (e.g., implementation by teachers and/or social workers), degree of incorporation into the formal curriculum (e.g., Life Education, Liberal Studies, Moral and Civic Education, Religious Studies and Integrated Humanities), use of different time slots (e.g., form teachers' period, morning assembly, weekly assembly, after-school period, Saturday mornings, extracurricular activities, and day camps), and combination of different teaching modes[11].

## Quality of Implementation

Despite the importance of quality of implementation in preventive programs, systematic research in this area is far from adequate[12,13]. Chen[14] provided a conceptual model for factors that influence implementation. These factors include characteristics of (1) the implementation system (i.e., process and structure of the implementation and training system), (2) the implementer (e.g., teacher and school staff), and (3) the setting in which the program is implemented (e.g., school climate, principal's support, and district support). In a comprehensive literature review, Shek and Sun[15] summarized five factors that influence the quality of program implementation, namely program, people, process, policy, and place (5 "P"s). These factors are examined in this case study.

The first element is a good **program**. The program should have the following attributes: well designed with clear goals; tailor made for adolescents' needs; socially and culturally sensitive; user friendly; with ready-made teaching materials; emphasis on practical competencies and generic skills; evidence-based program design; design conducive to the instructors' willingness to implement the program as intended, without overlapping with other existing programs in the school and with novel and attractive activities[12,16,17,18].

Second, **people** are crucial to the effective implementation of school-based prevention programs[12,14,19]. Research studies showed that the attributes include the leadership of the school principal[20]; committed coordinators who offer ongoing support to the front-line teachers, fostering communication among different parties, and providing clear guidance and information regarding the program implementation[12]; and training workshops for teachers to familiarize themselves with the program philosophy and content, the necessary teaching strategies, and the need for fidelity, as well as cultivating their enthusiasm and support for the program[12,21]. Research studies also showed that characteristics of the teachers, including rich teaching experience, good preparation for teaching, possession of a graduate degree, and a greater sense of belonging to the school contributed to program effectiveness[18,22].

In terms of the school-based prevention program implementation **process**, seven attributes were found in research studies[12,18,19]. They are (1) using interactive teaching strategies, (2) using facilitation skills to teach the program concepts and skills so as to generalize the students' learning, (3) adherence to the designed program, (4) good classroom management skills, (5) good time management skills, (6) support of the principal in the implementation process, and (7) enthusiastic teachers who create a positive classroom atmosphere that raises the students' learning interest and motivation.

Concerning school **policy**, elements of effective program implementation include integrating the program into the school formal curriculum, emphasizing program fidelity, existence of clear goals in the program implementation process, and good manpower deployment with division of labor to lessen the load of teaching and administrative duties[12,20]. Researchers also suggested that the school arrange regular meetings with teachers to discuss problems encountered and the respective solutions, and to build a supportive working environment as well as organize final evaluation meetings to applaud good work done, review past practices, and gain feedback[12]. Darden[23] elucidated that a good school policy is easy to understand, reasonable to comply with, and hard to misinterpret. He described the four essential steps to policymaking: (1) adoption; (2) implementation; (3) enforcement; and (4) periodic update. According to Darden[23], school policies need to be realistic, state clearly the school's expectations, and the schools should not expect the impossible and must align policies with available human and financial resources.

Finally, a warm and supportive school environment ("**place**"), where the school principal, coordinators, and teachers have the same view for supporting the implementation of the program in the school, is conducive to effective program implementation. Chen[14] argued that although an intervention is the major change agent in a program, the "implementation system" is likely to make an important contribution to program outcomes. The implementation system provides the means and a context for the intervention, and is affected by a number of factors, including characteristics of the implementers (i.e., "people" in this article), the nature of the implementing organization, and the quality of the linkages between this organization and the broader community[14]. Research findings supported that a nonauthoritarian working environment within the school that allows collaborative decision making and a positive atmosphere that enables job satisfaction will facilitate effective program implementation[22,24]. Furthermore, Eccles and Gootman[25] listed some physical features that promote positive youth development, including settings characterized with physical and psychological safety, appropriate structure, supportive relationships, opportunities to belong, positive social norms, support for efficacy, opportunities for skill building, and integration of family, school, and community efforts.

The strengths of case study research have been identified in the literature[20,26,27]. In this study, a case study was conducted in order to understand the contribution of the above factors to the quality of program implementation in the Chinese culture. There were two objectives of the case study: (1) to investigate the facilitating factors and obstacles encountered in the program implementation process from the program implementers' perspectives and (2) to draw implications for future program implementation. Specifically, Chen's[14] conceptual model for three key areas (i.e., the implementation system, the implementer, and the setting in which the program is implemented) was adopted in order to examine the case in light of the five categories of factors (i.e., program, people, process, policy, and place) that influence program outcomes.

## METHODS

### Participants

There were 207 schools that joined the Project P.A.T.H.S. (Secondary 1 Curriculum) in the Full Implementation Phase in the 2006/07 academic year. In the Tier 1 Program, 112 of these schools adopted the full program (i.e., 20-h program involving 40 teaching units) and 95 adopted the core program (i.e., 10-h program involving 20 teaching units). Among these participating schools, six schools that joined the full program and one school that joined the core program were invited to participate in the case study. Based on the convenience sampling method, one school where the Tier 1 Program was integrated into the subject of Liberal Studies was selected in this case study.

### Procedures

In this case study, a group interview with the school contact person and the vice-principal, and a focus group interview with three teachers of the Tier 1 Program of the Secondary 1 Curriculum were conducted separately. Prior consent from the principal was sought for the school's participation. At the beginning of the interviews, informed consent was also obtained from the respondents, and the purpose of the study and the principle of confidentiality were repeatedly emphasized. The author and a trained colleague with a Bachelor degree in Social Work conducted the interviews. As the purpose of this case study was to examine the school administrative arrangement and its implementation process of the Tier 1 Program (Secondary 1 Curriculum) in a retrospective manner, the interviews were conducted in early July 2007 after the school had completed the Tier 1 Program. Specific self-constructed semi-structured interview guides for the interview and the focus group interview were designed to collect the data. There were two parts in each interview guide:

- Respondents' perceptions of the school administrative arrangement, including preparation, support within the school and from the social work agency, and program evaluation
- Respondents' perceptions of the program implementation process

In addition, the school was required to complete a school background information questionnaire with both closed- and open-ended questions in order to provide basic information about the school, the Tier 1 Program implemented in the school, instructors conducting the Tier 1 Program, and the implementation process.

After data collection, the data were neatly typed and the recordings of the interviews were transcribed. Before data analysis, the raw transcription scripts were sent to the interview participants for their confirmation. No error was found in the precision of the dialogue in the transcribed texts. The procedures for data collection described above satisfied Principle 3 in the implementation of evaluation research proposed by Shek et al.[28].

### Data Analyses

The qualitative data were analyzed using general qualitative analyses techniques[29]. There were three steps in the process. First, words, phrases, and/or sentences that formed meaningful units at the raw response level were coded. Second, with reference to the questions of the interview guide, the codes were further combined to reflect higher-order attributes at the category of codes level. Third, the categories of codes were further analyzed to reveal the broader themes at the thematic level. Following the principles of qualitative analyses[28], the raw data and categorized data were kept in a systematic filing system for easy reference and audit.

## RESULTS

### Summary of the School Characteristics

The interviewed school participated in the Tier 1 Program (Secondary 1 Curriculum) in the Full Implementation Phase in the academic year 2006/07. As shown in Table 1, there were 215 Secondary 1 students in five classes. The 20-h full program (20 1-h sessions) was integrated and delivered in Liberal Studies lessons (20 h) from January to March 2007 and project-based learning. In addition, a teaching activity day was organized for all Secondary 1 students in which demonstrations and a large-scale activity were run by the social workers with the participation of the teachers who taught Secondary 1. A conclusion session was also arranged for students in the format of whole-form gatherings after the program implementation. The medium of instruction was Chinese. Although the school had a double class teachers system, only the teacher who teaches the Liberal Studies lessons implemented the program with the assistance of a teaching assistant. The school had also made prior arrangement with the school of education of a local university to assign a student-teacher under internship to join in some of the lessons

**TABLE 1**
**Summary of the Characteristics of the School**

| | |
|---|---|
| **I. Basic Information of the School** | |
| Total no. of S1 students (no. of S1 classes) | 215 (five classes) |
| Class teacher system | Double class teachers system |
| Participation in Life Education program organized by other organizations | Yes (Basic Life Skills Training Program, Department of Health) |
| No. of uniform/social and voluntary services groups in school | No |
| Small groups, workshops, and seminars specifically arranged for S1 students | (i) Antidrugs; (ii) Mental health; (iii) S1 orientation; (iv) Proschool culture; and (v) Religious groups. |
| **II. Basic Information of Tier 1 Program Participation** | |
| Hours | 20-h full program |
| Mode | Mode 3 (20 1-h sessions) |
| Program arrangement | Liberal Studies lesson (100%) |
| Rationales of the program arrangement | P.A.T.H.S. topics related to individual aspects of Liberal Studies. |
| Medium of instruction | Chinese |
| Consolidation session | N.A. |
| Beginning session | Yes (S1 orientation) |
| Conclusion session | Yes (growth puzzle) |
| Supplementary activity | Yes (project-based leaning) |
| Other activities | No |
| Student assessment on P.A.T.H.S. Program | Yes (homework) |
| Have joined Experimental Implementation Phase | No |
| **III. Information of the Instructors Conducting Tier 1 Program** | |
| No. of instructors who conducted Tier 1 Program | Three teachers, eight teaching assistants |
| No. of instructors who joined P.A.T.H.S. training | Five teachers |
| No. of instructors who joined the workshops on “Handling Behavioral and Emotional Problems of Students” | None |
| No. of instructors who joined the workshops on “Personal Growth and Psychological Well-Being of Teaching Professionals” | None |
| **IV. Basic Information of Tier 1 Program Implementation** | |
| No. of preparation meetings and content | Two preparation meetings for class teachers to discuss the aims, implementation arrangement, and related matters |
| No. of instructors who conducted Tier 1 Program in each class | One teacher plus one teaching assistant |
| Person responsible for preparing the teaching materials and the way of preparation | Class teachers |
| Format and frequency of lesson preparation among instructors | Once per week |
| Format and frequency of experiences sharing after class among instructors | Once per week |
| No. of evaluation meetings and content | One interim and one final evaluation meetings, mainly focusing on the level of student involvement and follow-up actions |

*Table 1 continues*

**TABLE 1 (continued)**

| | |
|---|---|
| Cooperation between the school and social work agency | Social work agency mainly responsible for the supplementary activity and Tier 2 program |
| The role of the teacher in Tier 1 Program implementation | (i) Teaching the program; (ii) Monitoring classroom discipline; (iii) Preparing teaching materials; (iv) Coordinating the program-related matters; (v) Follow-up. |
| The role of the social worker in Tier 1 Program implementation | Coordinating the program-related matters |

to provide help in small group discussions and running activities. In short, two to three persons cooperated in running the program in the Liberal Studies class periods. In total, three class teachers (two of them both taught two classes) were mainly responsible for the implementation of the Tier 1 Program in the school with eight teaching assistants and two subject teachers (one of them was a vice-principal) providing support. The school sent a total of five teachers to take part in the training workshops arranged for the instructors before the implementation of the program. Only three of them actually ran the program due to practical manpower deployment considerations.

The school arranged different kinds of meetings in the program implementation process. Before implementation, there were two preparation meetings, where the implementation objectives and arrangements were discussed. During the 3-month implementation period, class teachers' meetings were held every week, in which teachers shared the program contents and strategies of teaching. There were an interim evaluation meeting and a final evaluation meeting after implementation. The focus of these meetings was on the level of student involvement and follow-up actions.

In the planning stage, the coordinator (a teacher) played an important role to facilitate the subject team to decide on integrating the program into the formal curriculum of Liberal Studies. During the implementation process, the coordinator was responsible for preparing summaries of all teaching plans and materials, as well as briefing the teachers. When teachers received the teaching plans, they discussed them together before running the activities in the classes. In the Tier 1 Program, the roles of teachers included teaching the program, monitoring classroom discipline, preparing teaching materials, coordinating the program-related matters, and following-up tasks. The social worker was mainly responsible for coordinating the program-related matters, but she did not conduct any activities in classes.

As shown in Table 2, the findings on the school administrative arrangements and related issues were divided into several parts.

a. **Policy environment** — The vice-principal revealed that education reform provided a necessary policy environment for the school to make changes at both the junior and senior secondary levels. He strongly supported the implementation of the school-based curriculum approach, which provided much room for adoption of many of the curriculum units provided by the Project P.A.T.H.S.
b. **School policy** — The vice-principal also revealed that in the previous 2 years, the school started to adopt components of Liberal Studies in the junior secondary curriculum in anticipation of the curriculum change in the future. He acknowledged that the P.A.T.H.S. program fitted very well with the specific areas of personal development and interpersonal relationships, i.e., Module 1 of Liberal Studies. He worked with the school coordinator to plan for the changes and adopted school-based Liberal Studies for Secondary 1 students in 2006/07. He also appreciated the groundwork that the school coordinator had done in studying the possible adoption of the P.A.T.H.S. program into the curriculum of the school-based Liberal Studies. A decision was then made at the school management level with consultations from front-line teachers.

**TABLE 2**
**Consolidated Interpretations on the Administrative Arrangements and Related Issues in the School**

| Aspect | Consolidated Interpretations |
|---|---|
| (a) Policy environment | • Education reform provided a favorable policy environment.<br>• Implementation of school-based curriculum approach provided room for adoption of a new curriculum P.A.T.H.S. |
| (b) School policy | • Middle management planned for the changes in the curriculum of Liberal Studies to meet future need.<br>• Decision made to support the adoption of the P.A.T.H.S. program at the highest level with consultations from teachers. |
| (c) Provide structure and resources in the early planning stage | • Assignment of a coordinator who also served as the contact person.<br>• Recruitment of student-teacher majors in Liberal Studies to serve as teaching assistants.<br>• Deployment of teaching assistants to assist teaching.<br>• No extracurricular activities would be scheduled on every Monday for the whole school so that teachers could group together for meetings and preparation work. |
| (d) Construction of the curriculum content | • A committee decided on the selection of curriculum units of the P.A.T.H.S. program to be included in Liberal Studies.<br>• Special needs of the students were taken care of through workshops and seminars specifically arranged for S1 students. |
| (e) Support for the program and its rationales | • Support from the principal, the school coordinator/contact person who considered the program design of the P.A.T.H.S. program applicable, useful, and more effective than traditional teaching and learning methods for the subject.<br>• The teachers agreed that the program provided chances for the teachers to understand the students and enhanced student-teacher relationships, which was essential to the teaching and learning processes.<br>• The P.A.T.H.S. program was chosen partly because it offered attractive teaching and learning activities. |
| (f) Program arrangement, including its advantages and disadvantages | • Advantages: (i) provided a chance to support student's personal growth; (ii) gave sufficient support in terms of research results, reference materials, teaching tips, audio-visual materials, suggestions for teaching and learning activities; and (iii) the 3-day training for teachers provided briefings on the curriculum structure and sufficient psychological preparation for teachers.<br>• Disadvantage: teachers who were not familiar with interactive teaching methods suggested more training be provided during the 3-day training workshop. |
| (g) Manpower deployment | • The manpower distribution in Tier 1 program: (i) one coordinator responsible for the administrative and coordination work; (ii) two class teachers taught their own classes; (iii) one teaching assistant was provided per lesson; and (iv) student-teachers majoring in Liberal Studies were arranged to serve as teaching assistants. |
| (h) Facilities for implementation | • Special arrangement in the use of a large special room was made to meet the need for space in running some activities.<br>• Teachers could use the copying machine freely for reproduction of teaching materials. |

*Table 2 continues*

**TABLE 2 (continued)**

| | |
|---|---|
| (i) Support within school and from the social work agency | • An atmosphere of trust among the teaching staff was acknowledged.<br>• Tasks of the school coordinator: (i) explained the program contents to all relevant teachers; (ii) arranged all relevant teachers to participate in the P.A.T.H.S. training program; (iii) made curriculum decisions with different working groups to avoid duplication; and (iv) promoted cooperation among different committees within the school.<br>• The school social worker provided help in Tier 2 program.<br>• The school decided to make use of the active learning approach of the P.A.T.H.S. program to strengthen the student-teacher relationship. |
| (j) Impression of the overall administration | • The coordinator was satisfied with the existing manpower deployment arrangement. |
| (k) Advice for administration for the next academic year | • Teachers suggested measures for improvement in the overall administration.<br>• Teachers suggested changing the delivery mode of the P.A.T.H.S. curriculum to one class per week. |
| (l) Other aspects | • Periodic evaluation meetings on Mondays among Secondary 1 class teachers together with the school coordinator. |

c. **Provide structure and resources in the early planning stage** — The vice-principal pointed out that the school had been working hard to build the curriculum for Junior Secondary Liberal Studies one level per year starting in 2006. By 2009, the school should have finished the important and difficult task. He recognized that the ready-made program materials of the P.A.T.H.S. program served as important resources for the building process. He also praised that the P.A.T.H.S. program was more than an ordinary curriculum, as it could help students learn and grow personally and interpersonally. In terms of initiating a structure to facilitate the adoption, he emphasized that other subject and functional committees were kept informed of the development of school-based Liberal Studies so that a consensus could be reached from the staff to put in resources for the development of the curriculum. At the same time, a coordinator was assigned to assume the coordination and curriculum planning tasks together with the subject teachers. The school also offered no extracurricular activities on every Monday so that teachers could form learning groups (quality circles) for meetings and preparation work. The vice-principal also planned ahead to recruit student-teachers majoring in Liberal Studies to come to the school to serve as teaching assistants. On top of these teaching assistants, the administration also deployed existing teaching assistants to assist teaching.

d. **Construction of the curriculum content** — The school contact person revealed that the committee on Liberal Studies decided to adopt the curriculum units of the P.A.T.H.S. program in school-based Liberal Studies. A teacher was assigned to serve as the coordinator who would study the curriculum and brief the teachers who would teach the subject. Special needs of the students were taken care of through workshops and seminars specifically arranged for S1 students.

e. **Support for the program and its rationales** — The vice-principal pointed out that the program was well designed to meet the special needs of his students who lived in a relatively deprived district. He highly commended the program that could help the students whose families could not provide such opportunities. The school contact person echoed that he considered the program to be especially helpful to those adolescents who would face a high level of frustration in the globalized economy. He strongly supported the program and agreed to its rationales, and thus he took up the responsibility to arrange for the program implementation in his school. He also pointed out that the good reputation of the program and the training also generated support among teachers. Similar support from teachers was also observed. They considered the program design of the Project P.A.T.H.S. to be practical, useful, and more effective than traditional teaching and learning methods for the subject. The teachers agreed that the program provided chances for the teachers to understand their students and enhanced student-teacher relationships, which were essential to the teaching and learning process.

f. **Perceived program arrangement, including its advantages and disadvantages** — The vice-principal pointed out three advantages of incorporating the program into the subject of school-based Liberal Studies: (i) the program provided a chance to support the student's personal growth; (ii) the project team gave sufficient support in terms of research results, reference materials, teaching tips, audio-visual materials, as well as suggestions for teaching and learning activities; and (iii) the 3-day training for teachers provided briefings on the curriculum structure and sufficient psychological preparation for teachers who would assume the teaching of the subject in the future. The school contact person appreciated the opportunity for the students to learn about the subject matter and he could not find any disadvantage of such an arrangement. This view was shared by other teachers in the focus group, except that one teacher suggested the need to spread the sessions over regular intervals throughout the year instead of compressing them within a short period of time. Another teacher who was not familiar with interactive teaching methods suggested more specific training on classroom management skills and in running facilitative activities during the 3-day training workshop.
g. **Manpower deployment** — From the interviews, it was revealed that the school had mobilized extra manpower, i.e., teaching assistants and student-teachers, to support the implementation of the program. The school contact person perceived that different teachers could complement one another through the learning group on the subject and the mutual support among group members was part of the school culture.
h. **Facilities for implementation** — As revealed by the teachers, the facilities for program implementation in each classroom were sufficient. Special arrangement for the use of a large special room was made to meet the need for space in running some activities. Teachers could use the copying machine freely for reproduction of teaching materials.
i. **Support within school and from the social work agency** — As revealed from the interviews, there were several aspects of support within the school and from the social work agency in the implementation process. The above findings indicated that (i) a trusting and supportive atmosphere among the teaching staff was acknowledged, (ii) tangible and intangible support from the vice-principal was evident, (iii) curriculum decision making involved different parties to avoid duplication, (iv) cooperation among different committees within school was promoted, and (v) the school social worker provided help in the Tier 2 Program.
j. **Impression of the overall administration** — The school contact person felt that he was satisfied with the administrative arrangement and communication among colleagues. The vice-principal also appreciated that the school contact person had followed through the program implementation diligently. Teachers were satisfied with the overall program administration.
k. **Advice for administration for the next academic year** — It was evident that good preparation and coordination work before the program implementation were needed. Teachers suggested changing the delivery mode of the P.A.T.H.S. curriculum to one class per week for the next year so that students can better comprehend the contents. Teaching assistants should be continuously provided because of the nature of the curriculum.
l. **Other aspects** — Meetings within school: In the interview with the school contact person, he revealed that there were periodic meetings of the learning group held on Mondays and a final evaluation meeting. These factors contributed to the smooth implementation.

## Implementation Characteristics in the School

The integrated findings and interpretations of the implementation characteristics are shown in Table 3.

**TABLE 3**
**Consolidated Interpretations on the Implementation Characteristics in the School**

| Aspect | Consolidated Interpretations |
|---|---|
| (a) Teachers' and students' responses in the implementation process | • The coordinator emphasized and praised students' participation in the class. |
| (b) Coteaching | • The school arranged teaching assistants to help run the activities, group discussions in the class. |
| (c) Teaching techniques | • Technique 1: Promoting trustful teacher-student relationships<br>• Technique 2: Promoting mutual understanding<br>• Technique 3: Solving problems in learning groups (Quality Circle) formed by teachers of the same subject<br>• Technique 4: Sensitive to the needs of students, e.g., showing appreciation, such as giving a round of applause or giving out sweets as a reinforcement. |
| (d) Handling students' worksheets | • There were no difficulties in worksheet preparation. |
| (e) Student assessment in the Tier 1 Program | • No assessment and examination for P.A.T.H.S. program units.<br>• Independent assessment for the subject of Liberal Studies. |
| (f) Difficulties encountered and solutions | • Difficulties encountered: (i) obtaining students' trust while maintaining classroom discipline; (ii) lack of manpower and space; (iii) some similar content found in the BLST (Basic Life Skill Training Program) and the P.A.T.H.S.; (iv) the school social worker could not provide help in running the activities because the school adopted the "cycle-system" in which different classes took the same subject at the same time.<br>• A solution for the first difficulty was to make use of the learning group held on Monday.<br>• No solution was mentioned for the rest. |
| (g) Advice for implementation in the next year | • To provide resources and teaching assistants continually. |

a. **Teachers' and students' responses in the implementation process** — The vice-principal revealed that he put much emphasis on the quality of the students' involvement in the learning process and he was satisfied with this. About the students' responses in the implementation process, the teachers pointed out that the students were encouraged to participate actively in class activities and small group discussions in particular. Some students might have been sidetracked while others participated diligently. The teachers also pointed out that the program offered a good chance for the teachers to understand their students. The contact person revealed that some students who sought attention would later know the limits and would reduce their annoying behaviors as the teacher showed a high level of acceptance. The teachers also pointed out that the students liked the audio-visual materials, activities, debates, and role play. However, for the class with many students who demonstrated maladjusted behaviors, the students were not open to sharing with the teachers, and some of them were not involved in the lessons. The teachers also pointed out that students were interested in novel topics and they felt bored when attending intensive sessions. Despite this, the coordinator reiterated the importance of students' active participation and praised their participation.
b. **Coteaching** — The focus group interview findings showed that the teaching assistants were supportive to the teachers and provided much help in the running of the activities, group discussions in the class.
c. **Teaching techniques** — Several techniques were used by the teachers in the program implementation process. In the interviews, all interviewees emphasized the need for trustful teacher-student relationships and understanding between the teacher and the students. How to promote such a relationship was one of the important topics for sharing among teachers. The teachers pointed out that it was also important for them to share ways of handling problems in classroom management in the learning group (Quality Circle) formed by teachers of the same subject. They agreed that teachers had to be sensitive to the needs of students and to show appreciation, such as giving a round of applause or giving out sweets as reinforcement.

d. **Handling students' worksheets** — The teachers felt that there were no difficulties in worksheet preparation, except for the class with many students who displayed maladjusted behaviors.
e. **Student assessment in the Tier 1 Program** — The school contact person reported that there was no formal assessment on the P.A.T.H.S. program, but there were assessments in the project-based learning for Liberal Studies.
f. **Difficulties encountered and solutions** — From the findings, the vice-principal gave an account of how the school made use of the changes in the policy environment and the availability of the resources provided by the P.A.T.H.S. program to facilitate a smooth adoption of the program or, in other words, a successful implementation. He did not perceive any big difficulties as he could provide structure and resources to support the move. The contact person had been to the training workshops, was familiar with the program, and was enthusiastic about the implementation. However, it was noted that the front-line teachers encountered more difficulties in obtaining students' trust while maintaining classroom discipline, facilitating discussion in small groups for a class with over 40 students, and nonparticipation in the training workshops might be one of the reasons for their frustration. Hence, support from the senior staff (in this case, the vice-principal), familiarization with the program, rich relevant teaching experiences, sufficient training, good preparation, and support among teachers were solutions to the difficulties encountered in the program implementation. In addition, attending training workshops was also important because teachers could familiarize themselves with the program rationales, content, and teaching strategies.
g. **Advice for implementation in the next year** — In the focus group interviews, the teachers suggested that the school should continue its effort to provide teaching assistants and teacher training. They also suggested measures for improvement in the overall administration and the need for expanding the teaching period to one session per week.

## DISCUSSION

The integrated interpretations presented above generate implications regarding future program implementation.

### Factors that Facilitate or Impede Program Implementation

With reference to the 5 "P"s (i.e., program, people, process, policy, and place) in the conceptual framework, factors that facilitate or impede program implementation are examined.

First, echoing previous findings, the present study showed that when the teachers perceived the program to be evidence informed with ample support from the organizer as well as the school, and the activities met the developmental needs of the students, they would support the implementation of the program in the school. Most importantly, the perceptions of having ready-made and helpful teaching aids and assistance were facilitating factors, whereas perceived difficulties in facilitating small group discussion in a class with over 40 students were the obstacles in program implementation.

Second, as the teachers are the actual implementers of the program, they have a very crucial role. This study showed that if the instructors were trained and familiar with the techniques of handling value-based issues, they would feel competent to implement the program, which in turn raised the implementation quality. In addition, this study showed that teachers' qualities, such as being passionate, responsible, skillful, and motivated to help students were key factors in gaining trust and respect from the students, which would enhance the implementation quality. Moreover, having supportive and cooperative colleagues was a crucial asset that enhanced the effective delivery of the program. Furthermore, this study showed that the use of appreciation would also enhance the quality.

Besides the qualities of teachers, the tangible and intangible support from the school principal and the coordinator were the main facilitating factors of program implementation quality. Finally, students were the key stakeholders in experiential learning. Results showed that students could be motivated by the teachers through building a trustful relationship, and showing an acceptance of individual differences would enhance the students' motivation and improve the learning atmosphere, and thus improve the implementation quality for students with special needs.

Third, the present findings suggest that effective implementation depends partly on the teaching techniques. Findings showed that students could be motivated to participate in discussion and presentation of their own views. Therefore, the use of interactive teaching strategies, facilitation skills, classroom management skills, and time management skills helped to create a positive learning atmosphere that raised the students' learning interest and motivation. The present study also suggests that value clarification skills are crucial as there is a distinct role in inculcating values in Liberal Studies. In the implementation of the program, values issues could not be avoided. Teachers with a strong background in information-based knowledge may find difficulties in facilitating discussions on values. Chatzifotiou[30] pointed out the differentiation between information-based knowledge and value-based issues, and argued for the clarification of the conceptually different goals in relation to the holistic development of a student. This division of roles creates difficulties when implementing the part on value-based issues of the curriculum. Furthermore, Chatzifotiou's study[31] showed that even though teachers perceived the long-lasting elements of the curriculum, they still lacked general knowledge and the teaching methods for delivering it. All these teaching techniques do not only facilitate teaching, but also strengthen the rapport between teacher and the students. In addition, good preparation and mutual support among colleagues can also raise the program implementation quality.

Fourth, to implement the program effectively, the school needs to have a clear policy regarding the practical arrangement and respective manpower deployment. The present findings suggest that the school had gone through Darden's[23] four essential steps to policymaking in schools and achieved favorable results. If a school can make use of the changes in the educational policy environment to facilitate school policy and structural changes, it is more likely that the school will pave the way for the success of the implementation of a new and "unfamiliar" curriculum.

Finally, it is necessary to have a supporting school culture in order to run a positive youth development program. The school can create space and time for the teachers to work together one day a week without extracurricular commitments so that they can communicate, share experiences, and form a learning group to support each other in the program implementation. In addition, the school could make use of available resources to provide necessary teaching assistance for teachers to carry out the activities, which can then lessen the teachers' workload.

## Implications of the Findings for Future Implementation of the Program

Based on the present findings and analyses, several implications for future program implementation were generated.

In the first stage, the school should consider a potential policy to adopt a positive youth development program and integrate it into an existing or a new subject. After careful examination of the curriculum with debates on its merits, the school may endorse the adoption and provide a structure and resources. During the second stage, the implementation task is to be managed by a coordinator with support from the subject teachers. In the third stage, the major emphasis is to support its smooth implementation. The teachers may form learning groups to facilitate mutual support. The next stage is for the school to have regular reviews to examine the implementation in a concentrated way and determine what parts need to be freshened up.

Schools may also need to address the following policy questions: (1) whether the committee has a systematic way of investigating and developing policies for consideration, (2) whether the school has clearly delegated authority to the coordinator to oversee enforcement of policy and to monitor the implementation quality, and (3) whether clear pathways exist for communicating policies to teachers, parents, and students.

The designed curriculum of the Project P.A.T.H.S. is knowledge based, culturally sensitive, with clear goals and objectives. It addresses the value issues by providing suggestions on teaching strategies and tips with pedagogical, psychosocial, and teaching quality criteria for flexible adoption into different cultural and situational backgrounds. However, the difficulties in facilitating discussion in small groups and clarifying values in a large class, as reported by a teacher, may well reflect the need to strengthen the training to help teachers handle value-based issues.

Echoing Domitrovich and Greenberg's[13] suggestion, it is necessary from the outset to make local adaptations when adopting programs for schools and additional changes will be made by local program deliverers over time. Each school has its own uniqueness in terms of student needs. Careful adoption and integration of the essential elements of a curriculum is also a necessary condition for successful implementation.

## Limitations of the Case Study

There are some limitations of the present study. First, as only one nonrandomly selected school was involved in the case study, generalization of the findings may not be desirable. However, this case study documented the school's successful experiences of the program implementation to provide food for thought for other schools. Second, as the present interviews only involved a vice-principal, a school contact person, and three subject teachers, the data collected would be more comprehensive if interviews with the school principal, teachers with special responsibilities to care for students' personal growth and special needs, form teachers, and the school social worker were involved. Due to the busy schedule and heavy workload, the intended interviewees could not attend the focus group. Third, the case study was conducted in a retrospective manner, and it would be more valuable if periodic interviews with the instructors and observations of the program delivery in classes could be conducted as well. However, that would then cause further interruption to the normal operation of the school. Fourth, in the data analyses, coding and categorization were done by a trained research assistant and cross-checked by the author. Although this method was justified for a small-scale case study, inter- and intrareliability tests were not conducted. Finally, the presence of interviewers may have affected the responses of the respondents (e.g., social desirability effect) in face-to-face interviews. However, as the transcribed texts were sent to the interviewees for review, this method of data collection was justified.

Despite the above limitations, the present findings are generally coherent with the evaluation findings generated from different sources and different methods, including objective outcome evaluation[32], subjective outcome evaluation[33,34,35,36], qualitative evaluation[37,38], process evaluation[39,40], and interim evaluation[11,41], which suggest that the Tier 1 Program (Secondary 1 Curriculum) of the Project P.A.T.H.S. is effective and well received by the different stakeholders of the program.

## ACKNOWLEDGMENTS

The preparation for this paper and Project P.A.T.H.S. were financially supported by The Hong Kong Jockey Club Charities Trust.
